# Inequality in fertility rate and modern contraceptive use among Ghanaian women from 1988–2008

**DOI:** 10.1186/1475-9276-12-37

**Published:** 2013-05-29

**Authors:** Benedict O Asamoah, Anette Agardh, Per-Olof Östergren

**Affiliations:** 1 Department of Clinical Sciences, Social Medicine and Global Health, Lund University, Malmö, Sweden; 2 Centre for Adolescent Health, Murdoch Children’s Research Institute, Royal, Children's Hospital, University of Melbourne, Victoria, Australia

## Abstract

**Background:**

In most resource poor countries, particularly sub-Saharan Africa, modern contraceptive use and prevalence is unusually low and fertility is very high resulting in rapid population growth and high maternal mortality and morbidity. Current evidence shows slow progress in expanding the use of contraceptives by women of low socioeconomic status and insufficient financial commitment to family planning programs. We examined gaps and trends in modern contraceptive use and fertility within different socio-demographic subgroups in Ghana between 1988 and 2008.

**Methods:**

We constructed a database using the Women’s Questionnaire from the Ghana Demographic and Health Survey (GDHS) 1988, 1993, 1998, 2003 and 2008. We applied regression-based Total Attributable Fraction (TAF); we also calculated the Relative and Slope Indices of Inequality (RII and SII) to complement the TAF in our investigation.

**Results:**

Equality in use of modern contraceptives increased from 1988 to 2008. In contrast, inequality in fertility rate increased from 1988 to 2008. It was also found that rural–urban residence gap in the use of modern contraceptive methods had almost disappeared in 2008, while education and income related inequalities remained.

**Conclusions:**

One obvious observation is that the discrepancy between equality in use of contraceptives and equality in fertility must be addressed in a future revision of policies related to family planning. Otherwise this could be a major obstacle for attaining further progress in achieving the Millennium Development Goal (MDG) 5. More research into the causes of the unfortunate discrepancy is urgently needed. There still exist significant education and income related inequalities in both parameters that need appropriate action.

## Introduction

Since the introduction of Millennium Development Goal 5 (MDG 5), the global community has seen some positive trends in maternal health, however changes are unevenly distributed [1-4]. Current evidence shows slow progress in expanding the use of contraceptives by women of low socioeconomic status [1] and insufficient financial commitment to family planning programs [5]. Modern contraception has played a key role in reducing the world’s total fertility rate, especially in developing countries [6-8]. Facilitating access to modern contraceptives for women with unmet needs for family planning has the potential benefit of improving maternal and child health and reducing mortalities [9-13] by lowering the annual number of unintended pregnancies [5]. However, differences exist in contraceptive use trends between and within countries [14], and this calls for examining country specific trends to guide local policy makers and interventionists on proper policies, resource allocations, and interventions that take into account the unmet needs of the most vulnerable women [15].

Inequity in health exists when people are unfairly deprived of the resources they need to maintain good health or protect themselves from unwanted or undesirable conditions [7]. The WHO commission on social determinants of health has redefined the unfair differences within and between groups as social injustice, which carries along with it moral implications. [16]. It is only through the equity lens that we can observe whether certain strata of the population, such as poor, young, single, rural residents and women with low educational background, are being deprived of the family planning resources needed to avoid unwanted pregnancies. However, the equity concept needs to be applied more cautiously when examining reproduction and the role of contraception compared to other health interventions [7]. Many existing studies that have examined health inequality within a population have only considered the wealth dimension [17,18]; however, this singular stratification is inappropriate. Studies that investigate inequality in health should examine the multiple dimensions of inequality that exist within countries such as age, residence (rural or urban), gender, marital status and educational level [17] based on the different human rights challenges, policy needs, and opportunities within a country. This is because the health gaps between these groups may be as significant as the gap between the rich and poor [17].

In most resource poor countries, particularly sub-Saharan Africa, modern contraceptive use and prevalence is especially low and fertility is very high resulting in rapid population growth and high maternal and child mortality and morbidity [11,12,19,20]. In sub-Saharan Africa, the trend in contraceptive use is divergent [17,21,22]. A study by Creanga *et al*. compared two Demographic and Health Surveys (DHS) in 13 sub-Saharan African countries. They reported a decrease in wealth-related inequalities in the met need for contraception in some countries and an increase in others. In a similar manner, it was found that contraceptive use in general increased substantially in Ethiopia, Madagascar, Mozambique, Namibia and Zambia attributable to the increased uptake of short-term contraceptive methods while there was a marginal decline in Malawi, Senegal and Uganda. Also, the increase in use of long-term contraceptive methods was almost negligible, and five countries (Cameroon, Kenya, Mozambique, Rwanda and Senegal) experienced a reversal trend in use [14]. Duff Gillespie *et al.* studying data from 41 developing countries found the poorest quintile had a total fertility rate of 6, twice as high as that found in the wealthiest quintile. They suggest that reducing inequality in access to modern contraception will also reduce the inequality in fertility [7]. Hotchkiss and colleagues observed that the increased role of the private commercial sector in supplying modern contraceptives in Nigeria, Uganda, Bangladesh and Indonesia resulted in reduced inequality in modern contraceptive prevalence rates over time [23].

Ghana was one of the first countries in sub-Saharan Africa to adopt an explicit and comprehensive population policy in 1969 [24]. As a result, the Ghana National Family Planning Programme was launched in May 1970. The major focus was to lower the high rate of population growth and to facilitate economic growth. Unfortunately the programme achieved poor success; this is attributed to the provider-focused delivery strategy, coupled with poor institutional coordination [25]. The policy was revised in 1994 with the following goals: to reduce the total fertility rate (TFR) from 5.5 to 5.0 by the year 2000, to 4.0 by 2010, and to 3.0 by 2020 through increased contraceptive use; to increase the modern contraceptive prevalence rate to 28% in 2010 and 50% in 2020; and to achieve a minimum birth spacing of at least 2 years for all births by 2020 [26].

Results from the women’s health survey conducted in the capital city of Ghana, Accra, in 2003 found that women’s education status was significantly associated with the odds of currently or ever using contraception [27]. Evidence from the Ghana Demographic and Health Survey (GDHS) 2008 indicates that there is almost universal knowledge of some form of contraceptive method, with 98% of all women and 99% of all men knowing at least one method of contraception. The use of modern methods of contraception increased more than three fold (from 5% to 17%) between 1988 and 2008, and in the same period Ghana’s TFR dropped from 6.4 to 4.0, putting Ghana among the lowest countries in sub-Saharan Africa [28]. These positive trends raise the question of equality: do all sub-populations in Ghana have equal access to modern contraceptives? Are some sub-populations deprived from accessing effective modern contraception? Is the inequality gap in the use of modern contraceptives, and fertility as a related outcome, increasing or decreasing in the population? This crucial knowledge is lacking from previous studies on modern contraception and fertility in Ghana. These are the questions that this current research seeks to address by examining the gaps, trends and patterns in modern contraceptive use and fertility within different socio-demographic subgroups in Ghana from 1988 to 2008.

## Methodology

### Data Collection

We constructed a database using data from the Ghana Demographic and Health Survey 1988, 1993, 1998, 2003 and 2008 carried out by the Ghana Statistical Service and the Ghana Health Service. These surveys employed standard DHS questionnaires and techniques for data collection. All eligible women aged 15–49 were interviewed with the Women’s Questionnaire. Eligible women were defined as all women aged 15–49 who stayed in a selected household the night before the interview, irrespective of whether they were usual residents in the household or not. The Women’s Questionnaire was used to collect information on the following topics: respondent’s background characteristics; reproductive history; contraceptive knowledge and use; antenatal, delivery and postnatal care; infant feeding practices; child immunization and health; marriage; fertility preferences and attitudes about family planning; husband’s background characteristics; women’s work; knowledge of HIV/AIDS and STDs; as well as anthropometric measurements of children and mothers. The study was done in compliance with the Helsinki Declaration.

### Variables

#### Definition of variables

Two outcome variables were used to assess the trend in family planning use.

1. *Non-use of modern contraceptive methods*. This variable was generated from the response to the question “ever used any method of family planning?” The responses were dichotomized into: ‘ever used modern method of contraception’ and ‘never used any modern method of contraception’. When a woman also used a traditional method, she was put into the first category.

2. *Fertility rate*. This variable was generated from response to the question that assessed the total number of children a woman had ever given birth to. The responses were re-categorized as: a) low fertility rate: having less than 4 live births and b) high fertility rate: having 4 or more live births.

#### Independent variables

The socio-demographic variables used in this study were as follows:

1. *Maternal age.* This variable was categorized into three age groups (less than 25, 25–34 and 35 or more)

2. *Educational level*. This was classified in four categories:

a. Never attended school (women who confirmed having no formal education)

a. Basic education (women with some level of formal education not exceeding nine years, including those with primary, middle school, or lower secondary school education)

a. Senior high school (women with up to 12 years of formal education or those whose education ended at the upper secondary school level)

a. Tertiary or higher education (women who completed at least 15 years of formal education, including those with college, polytechnic, or university level studies)

3. *Residence*. Residence was coded as either urban or rural

4. *Current marital status*. Current marital status was classified in two categories:

a. Single (women who had never married, or were separated, divorced, or widowed at the time of the interview)

a. Married (women who were married or living with a partner at the time of the interview)

5. *Income level*. Income level was calculated based on the yearly earnings of the respondents. This variable was originally categorized into 5 quintiles (poorest, poorer, middle, richer, richest) according to the Ghana Demographic and Health Survey. They were later ranked into 3 groups: low income, average income and high income using the fractional rank function in SPSS.

### Statistical methods and analysis

#### Measures of Inequality

To measure health or health-related inequality in a population, there is a choice to use either the relative or absolute measure or both. Two frequently cited articles written by economists [29] and social epidemiologists [30] discuss both relative and absolute measures of inequality and arrive at complex measures argued to capture socioeconomic differences in health best [31]. Mackenbach and Kunst [30], while agreeing with Wagstaff *et al.*’s [29] choices (Concentration Index, Relative Index of Inequality and Slope Index of Inequality) as appropriate measures of health-related inequalities, also recommend other simple measures, such as the prevalence differences, and more sophisticated measures, such as regression based population attributable risk. They argue for and recommend the use of simple measures of inequality in health as well as methodologically more refined measures to complement each other. The theoretical foundations of both Kunst and Mackenbach’s Relative Index of Inequality, while accepted by several publications, have faced considerable criticism by the epidemiological community. Also, recent studies have stressed that relative measures of inequality need to be complemented by measures of absolute health inequalities such as prevalence differences and slope index of inequality [31].

In this current study, we mainly applied regression-based Total Attributable Fraction (TAF) [32] which we consider more robust in measuring inequalities in health. We also calculated the Relative and Slope Indices of Inequality (RII and SII) to complement the TAF. The statistical software IBM SPSS Statistics 20 and Microsoft Excel were used for analysis.

##### Total Attributable Fraction (TAF)

TAF represents the proportion of the outcome that would not exist if all women had the same prevalence as those with highest socioeconomic status, under the assumption that there is a causal pathway between socioeconomic status and the outcome variable. The attributable fraction was calculated using the formula *AF* = (*OR* − 1)/*OR*, where OR is the adjusted odds ratio generated from the logistic regression analysis. Total attributable fraction (TAF) was calculated as follows: *TAF* = ∑ (*sTAF*) = ∑ *AF*_*i*_ * *P*_*i*_ where AF_i_ is the attributable fraction for the outcome variable for a specific stratum and P_i_ represents the proportion of all cases that fall in this stratum. The Product of AF_i_ and P_i_ represents the stratum-specific Total Attributable Fraction (sTAF), and ∑ (*sTAF*) indicates the summation of all the strata-specific calculations, referred to as the overall TAF. For those with the highest level of education, the AF and sTAF are by definition zero.

##### Relative Index of Inequality (RII)

The extent of inequality is easier to interpret when it is expressed as Relative Index of Inequality, defined by Mackenbach and Kunst. However, when using a categorical outcome, the Relative Index of Inequality is based on odds ratio, making its interpretation more complex. This conflicts with our desire to express the magnitude of inequalities in concrete terms that can be understood and interpreted by a broad audience. We therefore applied a more refined method of calculating Relative Index of Inequality proposed by Koolman and colleagues which is based on relative risk rather than odds ratio [31,33]. This method used estimates derived from the logistic regression to compute relative risks between rural and urban residence, and relative indices of inequality based on ranking all individuals according to income and education. Highest educational level was converted to a numerical measure by ranking it between 0 and 1. The ranked education and income variables were then entered into a logistic regression model as a continuous covariate with ‘*never used any modern method of contraception’* or ‘*four or more live births’* as the outcome. Additional adjustment was made for age, marital status and rural/urban residence status.

The procedure for computing the RII [31] was as follows:

1. Estimate the logistic regression and retain its coefficients.

2. Predict the outcome for each group while fixing the category of interest at one.

3. Repeat step 2 but now fixing the dummy or rank at zero.

4. Divide the average outcome of step 2 by the average outcome of step 3.

For dichotomous variables (rural/urban residence status), this produces relative risk (interpreted like the RII) directly comparable to the odds ratio produced by the logistic regression. For ranked variables (education and income level) the above procedure produces a relative index of inequality based on relative risk and can be interpreted as the relative risk of each individual reporting an outcome had she moved from the very highest to the very lowest rank.

##### Slope Index of Inequality (SII)

To obtain the SII, the result from step 3 above is subtracted from those of step 2. The SII can be interpreted as the absolute difference in the probability of reporting an event between the group/person with the lowest rank and the highest rank.

## Results

The number of respondents to the Women’s Questionnaire and response rates were 4488 (response rate 98%) in 1988, 4562 (response rate 96%) in 1993, 4843 (response rate 97%) in 1998, 5691 (response rate 96%) in 2003 and 4916 (response rate 97%) in 2008. About one third of the women had high fertility rates in 1988 and this proportion decreased gradually but consistently over the 20 years period. During the same period, there was a parallel decrease in non-use of modern contraceptive methods (Table 1).

**Table 1:** Socio-demographic characteristics, fertility rate and modern contraceptive use and valid per cent (%) among Ghanaian women 15–49 years from 1988 to 2008 presented in 5 years intervals

**Variables**	**Year**				
	**1988**	**1993**	**1998**	**2003**	**2008**
**Age**					
<25	1716(38.2)	1632(35.8)	1776(36.7)	2110(37.1)	1906(38.8)
25-34	1511(33.7)	1588(34.8)	1518(31.3)	1784(31.3)	1453(29.6)
35+	1261(28.1)	1342(29.4)	1549(32.0)	1797(31.6)	1557(31.7)
**Total**	**4488**	**4562**	**4843**	**5691**	**4916**
**Residence**					
Urban	1523(33.9)	1720(37.7)	1585(32.7)	2374(41.7)	2162(44.0)
Rural	2965(66.1)	2842 (62.3)	3258(67.3)	3317(58.3)	2754(56.0)
**Total**	**4488**	**4562**	**4843**	**5691**	**4916**
**Educational level**					
Never attended	1783(39.7)	1597(35.0)	1737(35.9)	1917(33.7)	1243(25.3)
Basic education	2369(52.8)	2497(54.7)	2636(54.4)	3156(55.5)	2892(58.9)
Secondary	296(6.6)	396(8.7)	365(7.5)	474(8.3)	596(12.1)
Higher	40(0.9)	72(1.6)	105(2.2)	144(2.5)	181(3.7)
**Total**	**4488**	**4562**	**4843**	**5691**	**4912**
**Marital status**					
Married	3156(70.3)	3204(70.2)	3229(66.7)	3694(64.9)	2950(60.0)
Single	1331(29.7)	1358(29.8)	1614(33.3)	1997(35.1)	1966(40.0)
**Total**	**4487**	**4562**	**4843**	**5691**	**4916**
**Income level**					
Low income	909(40.4)		1323(41.4)	2338(41.1)	2010(40.9)
Average income	474(21.1)		630(19.7)	990(17.4)	897(18.2)
High income	868(38.6)		1240(38.8)	2363(41.5)	2009(40.9)
**Total**	**2251**		**3193**	**5691**	**4916**
Missing	2237	No data	1650	0	0
**Fertility rate**					
Low (less than 4 live births)	2738(61.0)	2909(63.8)	3201(66.1)	3812(67.0)	3458(70.3)
High (4 or more live births)	1750(39.0)	1653(36.2)	1642(33.9)	1879(33.0)	1458(29.7)
**Total**	**4488**	**4562**	**4843**	**5691**	**4916**
**Use of Modern contraceptive method**					
Ever used a modern method	918(20.5)	1344(29.5)	1424(29.4)	2073(36.4)	2001(40.7)
Never used any modern method	3570(79.5)	3218(70.5)	3419(70.6)	3618(63.6)	2915(59.3)
**Total**	**4488**	**4562**	**4843**	**5691**	**4916**

Within the 20-year period, the prevalence of non-use of modern contraceptive methods decreased, although unequally among all socio-demographic subgroups. Among women with no- and basic educational levels, the decrease in prevalence of non-use of modern contraceptive methods were more pronounced compared to those with secondary and higher educational levels. Within the income bracket, women with average income levels had the highest reduction in the prevalence of non-use of modern contraceptive methods (a prevalence difference of 18.2%), followed by those with low income (a prevalence difference of 14.5%) and high income levels (a prevalence difference of 11.0%) (Table 2).

**Table 2:** Prevalence, adjusted odds ratio and 95% confidence interval of non-use of modern contraceptive method according to socio-demographic characteristics among Ghanaian women 15–49 years from 1988 to 2008 presented in 5 years intervals

**Variables**	**Non-use of modern contraceptive method**				
	**1988 n (%) aOR(95% CI)**	**1993 n (%) aOR(95% CI)**	**1998 n (%) aOR(95% CI)**	**2003 n (%) aOR(95% CI)**	**2008 n (%) aOR(95% CI)**
**Residence**^**a**^					
**Urban**	1102(72.4) Ref	1047(60.9) Ref	994(62.7) Ref	1369(57.7) Ref	1180(54.6) Ref
**Rural**	2468(83.2) 1.4(1.2-1.8)	2171(76.4) 1.5(1.3-1.7)	2425(74.4) 1.2(1.0-1.4)	2249(67.8) 1.1(0.9-1.3)	1735(63.0) 1.1(0.9-1.3)
**Total**	**3570**	**3218**	**3419**	**3618**	**2915**
**Educational level**^**a**^					
**Never attended**	1598(89.6) 6.6(3.1-14.2)	1387(86.9) 14.9(8.5-25.6)	1424(82.0) 5.2(3.2-8.5)	1452(75.7) 4.1(2.8-6.0)	856(68.9) 3.9(2.7-5.5)
**Basic education**	1783(75.3) 2.1(1.0-4.4)	1608(64.4) 3.4(2.0-5.7)	1744(66.2) 2.2(1.3-3.4)	1834(58.1) 1.5(1.0-2.1)	1663(57.5) 1.8(1.3-2.5)
**Secondary**	174(58.8) 1.1(0.5-2.5)	202(51.0) 2.0(1.1-3.5)	209(57.3) 1.7(1.0-2.9)	265(55.9) 1.2(0.8-1.8)	325(54.5) 1.3(0.9-1.9)
**Higher**	15(37.5) Ref	21(29.2) Ref	42(40.0) Ref	67(46.5) Ref	69(38.1) Ref
**Total**	**3570**	**3218**	**3419**	**3618**	**2913**
**Income level**^**a**^					
**Low income**	736(81.0) 1.6(1.3-2.0)	No data	958(72.4) 1.5(1.2-1.8)	1664(71.2) 1.5(1.2-1.8)	1337(66.5) 1.3(1.1-1.6)
**Average income**	355(74.9) 1.3(1.0-1.7)		392(62.2) 1.1(0.9-1.3)	629(63.5) 1.2(1.0-1.5)	509(56.7) 1.0(0.8-1.1)
**High income**	557(64.2) Ref		699(56.4) Ref	1325(56.1) Ref	1069(53.2) Ref
**Total**	**1648**		**2049**	**3618**	**2915**

^*a*^*adjusted for age, marital status and mutually for one another*; *aOR = adjusted Odds ratio.*

The strength of association generally decreased for residence status, all levels of income and all levels of education with the exception of secondary education that recorded a slight increase.

From 1988 to 2008, there was a general decrease in the prevalence of high fertility within all socio-demographic subgroups, with the exception of women with no education who recorded no decrease in prevalence over the same period. Women in the higher education category recorded the highest prevalence reduction from 32.5% to 8.8% (a prevalence difference of 24.0%). Rural women recorded a decrease in prevalence of 4.7% whereas urban women recorded a decrease in prevalence of 13.3% over the same period. In the income level group, women with high income (top category) had the highest reduction in the prevalence of high fertility rate (a prevalence difference of 26.3%), followed by those with average income (a prevalence difference of 12.2%) and low income (a prevalence difference of 10.9%) (Table 3).

**Table 3:** Prevalence, adjusted odds ratio and 95% confidence interval of high fertility rate (4 or more live births) according to socio-demographic characteristics among Ghanaian women 15–49 years from 1988 to 2008 presented in years intervals

**Variables**	**High fertility**				
	**1988**	**1993**	**1998**	**2003**	**2008**
	**n (%) aOR(95% CI)**	**n (%) aOR(95% CI)**	**n (%) aOR(95% CI)**	**n (%) aOR(95% CI)**	**n (%) aOR(95% CI)**
**Residence**^**a**^					
**Urban**	509(33.4) Ref	432(25.1) Ref	370(23.3) Ref	527(22.2) Ref	434(20.1) Ref
**Rural**	1241(41.9) 1.4(1.1-1.7)	1221(43.0) 1.9(1.5-2.2)	1272(39.0) 2.0(1.7-2.5)	1352(40.8) 1.1(0.9-1.4)	1024(37.2) 1.4(1.1-1.8)
**Total**	**1750**	**1653**	**1642**	**1879**	**1458**
**Educational level**^**a**^					
**Never attended**	989(55.5) 4.5(1.8-11.1)	846(53.0) 6.2(3.2-12.1)	871(50.1) 8.8(4.8-16.2)	986(51.4) 3.8(2.2-6.6)	689(55.4) 7.9(4.3-14.3)
**Basic education**	719(30.4) 3.1(1.3-7.5)	739(29.6) 4.3(2.2-8.4)	711(27.0) 4.9(2.7-8.8)	828(26.2) 2.7(1.6-4.7)	714(24.7) 3.8(2.1-6.8)
**Secondary**	29(9.8) 0.6(0.2-1.6)	53(13.4) 1.4(0.7-2.8)	42(11.5) 2.0(1.0-4.0)	42(9.1) 1.0(0.5-1.9)	37(6.2) 1.3(0.7-2.6)
**Higher**	13(32.5) Ref	15(20.8) Ref	18(17.1) Ref	22(15.3) Ref	16(8.8) Ref
**Total**	**1750**	**1653**	**1642**	**1879**	**1456**
**Income level**^**a**^					
**Low income**	470(51.7) 1.0(0.8-1.3)		595(45.0) 1.1(0.9-1.4)	1033(44.2) 3.0(2.3-4.0)	821(40.8) 2.1(1.6-2.7)
**Average income**	202(42.6) 0.8(0.6-1.1)		265(42.1) 1.3(1.0-1.7)	365(36.9) 2.4(1.8-3.1)	273(30.4) 1.8(1.4-2.4)
**High income**	385(44.4) Ref		458(36.9) Ref	481(20.4) Ref	364(18.1) Ref
**Total**	**1057**	No data	**1318**	**1879**	**1458**

^*a*^*adjusted for age, marital status and mutually for one another*; *aOR = adjusted Odds ratio.*

The strength of association increased markedly for women with no formal education, basic education, low income and average income.

**Figure 1:**
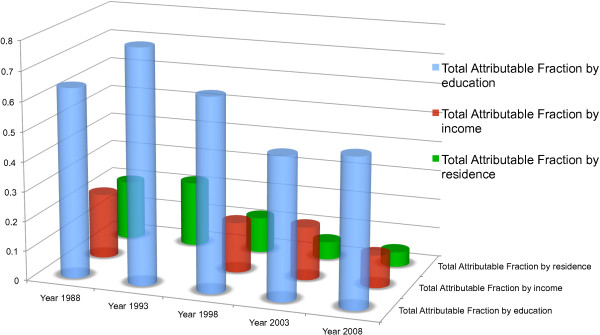
Comparing inequality in non-use of modern contraceptives by education, income and residence in Ghana.

The results generally show a decreasing trend in inequalities in non-use of modern contraceptive methods related to education, income and residence between 1988 and 2008 (Figure 1). Education-related TAF of non-use of modern contraceptive methods showed a marginal decrease (TAF = 0.64 in 1988 and 0.50 in 2008). Whereas sTAF for non-use of modern contraceptive methods decreased for women with no education from 0.38 in 1988 to 0.22 in 2008, that of women with basic education decreased very slightly from 0.26 to 0.25 over the same period. Income-related TAF decreased from 0.22 to 0.10 and that of residence decreased from 0.20 to 0.05 between 1988 and 2008. The corresponding Relative and Slope Indices of Inequality (RII and SII) followed a similar trend (Table 4).

**Table 4:** Logistic regression-based Attributable Fraction (AF), Stratum-specific Total Attributable Fraction (sTAF), overall Total Attributable Fraction (TAF), and Relative and Absolute Indices of Inequality (RII and SII) in Non-use of modern contraceptive method in each stratum from 1988 to 2008 presented in 5 years intervals

**Variables**	**Non-use of modern contraceptive method**				
	**1988**	**1993**	**1998**	**2003**	**2008**
**Residence**^**a**^					
Urban	Ref	Ref	Ref	Ref	Ref
Rural: AF (sTAF)	0.29(0.20)	0.33(0.22)	0.17(0.12)	0.09(0.06)	0.09(0.05)
Total Attributable Fraction (TAF)	0.20	0.22	0.12	0.06	0.05
Relative Index of Inequality (RII)	1.43	1.47	1.12	1.06	1.05
Slope Index of Inequality (SII)	9.78	8.05	0.89	0.62	0.33
*p*-value	0.001	<0.001	0.081	0.299	0.355
**Educational level**^**a**^					
Never attended: AF (sTAF)	0.85(0.38)	0.93(0.40)	0.81(0.34)	0.76(0.30)	0.74(0.22)
Basic education: AF (sTAF)	0.52(0.26)	0.71(0.36)	0.55(0.28)	0.33(0.17)	0.44(0.25)
Secondary: AF (sTAF)	0.09(0.004)	0.5(0.03)	0.41(0.03)	0.17(0.01)	0.23(0.03)
Higher	Ref	Ref	Ref	Ref	Ref
Total Attributable Fraction (TAF)	0.64	0.79	0.65	0.48	0.50
Relative Index of Inequality (RII)	11.32	18.86	6.00	6.63	4.55
Slope Index of Inequality (SII)	27.9	21.83	7.09	8.96	5.41
*p*-value	<0.001	<0.001	<0.001	<0.001	<0.001
**Income level**^**a**^					
Low income: AF (sTAF)	0.38(0.17)	No data	0.33(0.15)	0.33(0.15)	0.23(0.11)
Average income: AF (sTAF)	0.23(0.05)		0.09(0.02)	0.17(0.03)	0
High income	Ref		Ref	Ref	Ref
Total Attributable Fraction (TAF)	0.22		0.17	0.18	0.11
Relative Index of Inequality (RII)	2.11	No data	1.89	1.93	1.59
Slope Index of Inequality (SII)	16.12		4.00	5.08	2.58
*p*-value	<0.001		<0.001	<0.001	0.005

^a^ adjusted for age, marital status and mutually for one another.

There was an increasing trend in inequalities in fertility rates related to education, income and residence from 1988 to 2008. The overall TAF of high fertility rate for educational level differences increased from 0.72 in 1988 to 0.78 in 2008 whereas that of income differences increased from 0 in 1988 to 0.37 in 2008. For residence, the TAF increased from 0.21 in 1988 to 0.39 in 1998 and then dropped again to 0.20 in 2008. The related RII and SII for high fertility increased in line with the TAF patterns (Table 5).

**Table 5:** Logistic regression-based Attributable Fraction (AF), Stratum-specific Total Attributable Fraction (sTAF) and overall Total Attributable Fraction (TAF) of high fertility rate in each stratum from 1988 to 2008 presented in 5 years intervals

**Variables**	**High fertility rate (4 or more live births)**				
	**1988**	**1993**	**1998**	**2003**	**2008**
**Residence**^**a**^					
Urban	Ref	Ref	Ref	Ref	Ref
Rural: AF (sTAF)	0.29(0.21) 0.21	0.47(0.35) 0.35	0.50(0.39) 0.39	0.09(0.06) 0.06	0.29(0.20) 0.20
Total Attributable fraction(TAF)					
Relative Index of Inequality (RII)	1.15	1.33	1.33	1.07	1.14
Slope Index of Inequality (SII)	0.54	0.70	0.69	0.49	0.84
*p*-value	0.006	<0.001	<0.001	0.137	<0.001
**Educational level**^**a**^					
Never attended: AF (sTAF)	0.78(0.44)	0.84(0.43)	0.89(0.47)	0.74(0.39)	0.87(0.41)
Basic education: AF (sTAF)	0.68(0.28)	0.77(0.34)	0.80(0.35)	0.63(0.28)	0.74(0.36)
Secondary: AF (sTAF)	-	0(−)	0.50(0.01)	0(−)	0.23(0.01)
Higher	Ref	Ref	Ref	Ref	Ref
Total Attributable fraction (TAF)	0.72	0.77	0.83	0.67	0.78
Relative Index of Inequality (RII)	4.70	3.89	5.60	3.17	7.17
Slope Index of Inequality (SII)	3.24	2.08	2.30	4.82	5.89
*p*-value	<0.001	<0.001	<0.001	<0.001	<0.001
**Income level**^**a**^					
Low income: AF (sTAF)	0(−)	No data	0.09(0.04)	0.67(0.37)	0.52(0.29)
Average income: AF (sTAF)	-		0.23(0.05)	0.58(0.11)	0.44(0.08)
High income	Ref		Ref	Ref	Ref
Total Attributable fraction(TAF)	0		0.09	0.48	0.37
Relative Index of Inequality (RII)	1.09		1.29	5.95	3.52
Slope Index of Inequality (SII)	0.33		0.63	5.86	4.90
*p*-value	0.701		0.176	<0.001	<0.001

^a^ adjusted for age, marital status and mutually for one another.

Figure 1 compares education, income and residence related inequality trends in non-use of modern contraceptives.

**Figure 2:**
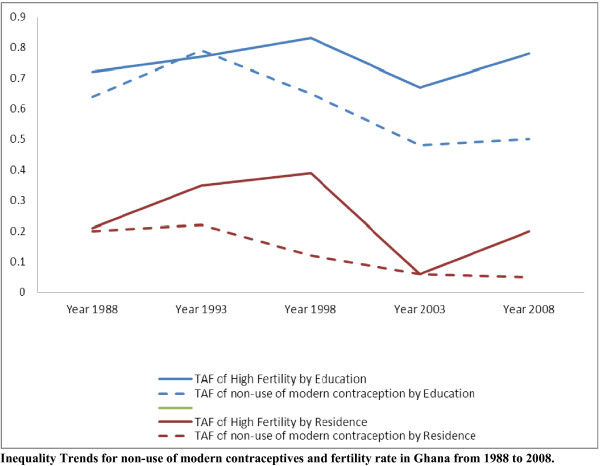
Inequality trends in non-use of modern contraceptives and fertility rate in Ghana between 1988 and 2008.

Figure 2 compares the inequality trends for non-use of modern contraceptives and fertility rate from 1988 to 2008.

**Figure 3:**
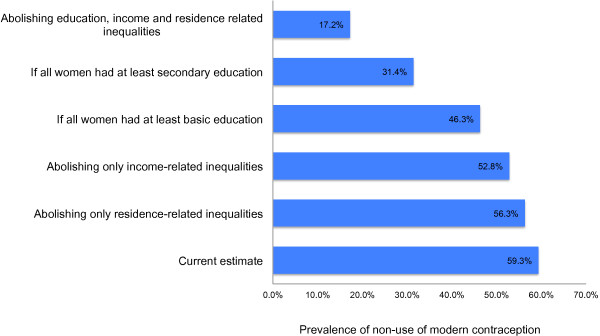
Implications of abolishing socioeconomic status related inequalities in non-use of modern contraceptives in Ghana in 2008.

Figure 3 provides an estimation of the implications of abolishing socioeconomic status related-inequalities in non-use of modern contraceptives in Ghana according to the current estimate.

## Discussion

### Summary of main results

The most important finding of this study was the observation that there has been a development towards equality in use of modern contraceptives, which is not mirrored in a similar trend in fertility (Figure 2). Contrary to the decreasing inequality trend observed in the use of modern contraceptives, this study showed an increasing trend in inequality in fertility rate related to education, income, and residence from 1988 to 2008. It is notable that the rural–urban residence gap in the use of modern contraceptive methods almost disappeared in 2008, while education and income related inequalities in the use of modern contraceptives still remained. There was also a consistent overall decrease in the prevalence of non-use of modern contraceptive methods among Ghanaian women, although it was unequally distributed among women with different socio-demographic backgrounds. During the same period (1988–2008), there was a parallel decrease in the proportion of Ghanaian women who had high fertility rate that was gradual but consistent over the period.

### Education-related inequalities in modern contraceptive use and fertility rate

It is essential to note that the overall increase in prevalence of modern contraceptive use observed between 1988 and 2008 was accompanied by a parallel decrease in the prevalence of high fertility rate over the same period. This corroborates findings from previous research on the essential role modern contraceptives play in fertility control [34]. Despite the reduced inequality in modern contraceptive use, our study also showed that inequality in fertility rate had increased over the 20 years period. This runs contrary to findings from Gillespie *et al.’s* study which suggested that reducing inequality in access to modern contraception will also reduce inequality in fertility [7].

Women with no formal schooling and basic education recorded a dramatic increase in the prevalence of modern contraceptive use. This widespread increase, seen in women of low educational backgrounds, is reflected in the sharp decrease in education-related inequality in modern contraceptive use between 1988 and 2008. Incidentally, this did not result in fertility decline within this group. Rather, a remarkable decline in fertility rate occurred in the highly educated women among whom contraceptive prevalence did not change much during the study period. A previous study conducted in Ghana between 1988 and 1998 found a moderate shift from using contraception to space pregnancies to its use for limiting childbirths, however they did not investigate differences within subgroups [34]. It is possible that women of different educational levels may use modern contraceptives for different reasons, be it for limiting or spacing childbirth. It can also be explained by the fact that women exposed to different levels of education may have different fertility preferences irrespective of their access to modern contraceptives. It may also be due to lack of access to information and services to prevent high-risk births which carries along with it moral implications in the case of unwanted fertility [7]. While fertility control can be enhanced by making modern contraceptives available and accessible to women [35], there are other factors beyond simple provision that influence women’s fertility rates or preferences [34]. For instance, highly educated women may have fewer children compared to less educated or illiterate women because of higher engagement and responsibilities in their professional careers [36] or during their education. In addition, method effectiveness could also play a role in the discrepancy between increased use of modern contraceptives and the unequal drop in fertility. Women with higher education may be more likely to choose more effective hormonal/long term methods whereas those with lower education may use more well-known/less effective methods like pills and condoms, both of which are classified as modern methods.

On aggregate, it is appreciable that the inequality gap in access to modern contraceptive methods by Ghanaian women decreased between 1988 and 2008 (Figure 1). That said, it is imperative to tackle the discrepancies that still exist between women with different educational attainments in Ghana with respect to their contraceptive use and fertility rate. From Table 3, it can be observed that low education is a significant predictor of high fertility rate and the strength of association doubles between 1988 and 2008. More so, the overall TAF for education increased within the same period from 0.72 to 0.78 indicating that in 2008 about 78% of women with a high fertility rate in Ghana would not have existed if all the women had education up to even secondary school level. It can be observed from Tables 4 and 5 that women with up to basic level of education were the sole contributors of the TAF for non-use of modern contraceptives and high fertility related to educational level differences. This implies that it has become more necessary now than ever to specifically address the needs of women with low education if there is to be real progress in target 5B of MDG 5, which aims to achieve universal access to reproductive health. This raises an issue of necessity for Ghana and other sub-Saharan African countries to look beyond MDG 2 (universal basic education). The attainment of universal basic education, according to current strategies and programs, will not necessarily lead to universal access and use of modern contraceptives with the potential outcome of lowering fertility rates in Ghana. Targeted programmes and policies on modern contraceptive use should be introduced that will specifically target the contraceptive needs of women with up to basic level of education [37]. This will help in addressing the education-related inequality gap that still exists in the use of modern contraceptive methods and fertility rates among Ghanaian women.

### Income-related inequalities in modern contraceptive use and fertility rate

Income-related inequality in the use of modern contraceptive methods has also reduced whereas that of fertility rate has increased. The reduced inequality in modern contraceptive use can partly be attributed to the availability of cost effective options over the years. Therefore, many more women in the low-income bracket in Ghana are able to afford the cost of modern contraceptives currently compared to previous years. For example, in 1988, 2.1% of the women gave high cost as the reason for non-use of contraception compared to only 0.7% in 2008 [38,39]. It is notable however that from 1988 to 1998, there were no significant differences in fertility rate between low, middle and high-income women. The differences became obvious and significant only after 1998 (Table 3). Between 1988 and 2008, the TAF for high fertility increased from 0 to 0.37 indicating that in 1988, if all Ghanaian women had the highest income level, it would not have made any difference in fertility rate. But currently (in 2008) about 37% of high fertility cases would not exist if all Ghanaian women were in the top income category. In Creanga *et al.’s* study looking at African women, it was found that women in the richest wealth quintile were more likely than those in the poorest wealth quintile to use long-term contraception after adjusting for fertility intentions. Long-term methods are more expensive and usually used to limit childbearing while short-term methods are normally suited for delaying but not forfeiting childbearing [14]. This may possibly provide a clue as to why income-related inequality in modern contraceptive use is decreasing against the increasing income-related inequality in fertility.

### Residence-related inequalities in modern contraceptive use and fertility rate

The downward trend observed in rural–urban residence related inequality in modern contraceptive use was very intriguing (Table 4). This is an indication that Ghana has progressed very well in reducing rural–urban inequalities in modern contraceptive use to almost zero. Much of the inequality in use that still exists among Ghanaian women is related to education rather than residence or income level. About 50% of non-use of modern contraceptives will be eliminated assuming every woman in Ghana is educated to secondary school level or higher, while only 5% of non-use will be eliminated if every woman in Ghana lived in an urban setting and only 11% if income differences were resolved (Figure 3). Again, this huge progress made towards equality in use of modern contraceptives did not translate into equality in fertility rate.

### Study limitations

A higher maternal mortality rate among women in low socioeconomic groups in the investigated population would bias the results towards the null. Thus, there is a possibility of underestimating the fertility rate by this method, since women who died before the interviews were excluded. If true, this method would most likely bias a “true” situation of higher fertility rates among lower socioeconomic groups towards the null. In other words, the true measure of inequality in fertility rate might have probably been underestimated. On the other hand, this is not expected to grossly affect the results of this study on aggregate since this is a trend analysis and its impact on change over time is minimal. That effect, even if present, will be considerably low since the lifetime risk of maternal death in Ghana is around 1.5%. Moreover, since infant and under-5 mortality is considerably higher among groups with low socioeconomic status, underestimation of high fertility in this group was eliminated by defining fertility based on the number of children a women ever had instead on the number of living children a woman had at the time of the interview.

There were high numbers of missing data on income in 1988 and 1998, and there was no data in 1993. This could have a probable effect on estimation of income related inequalities for 1988 and 1998 and also made it not possible to estimate the income related inequalities in year 1993. However, comparing the percentage distribution of income for 1988, 2003, and 2008, suggest that there is a fair distribution of women within the different income groups and that the overall effect of the missing data on the income-related inequality estimation will be minimal.

The current findings do not differentiate type of modern contraceptive which could possibly offer an evidence-based explanation for the discrepancy found in contraceptive use and fertility trends.

## Conclusion

One obvious observation is that the discrepancy between equality in use of contraceptives and equality in fertility must be taken very seriously and addressed in a future revision of relevant policy. Otherwise this could be a major obstacle for attaining further progress in achieving MDG 5. More research into the causes of the unfortunate discrepancy is urgently needed.

Our findings also indicate there still exists significant education and income related inequalities in both variables that need appropriate action if real progress is to be made in MDG 5B. The trends indicate that much of the inequality in the use of modern contraceptive methods that still exist among Ghanaian women is related to education rather than residence or income level and women with up to basic educational level are the most disadvantaged. Targeted programmes and policies on modern contraceptive use should be introduced in Ghana that will specifically focus on the contraceptive needs of women with up to basic level of education. Finally, education on fertility and modern methods of contraception should be taught in the basic schools in Ghana.

## Competing interests

The authors declare that they have no competing interests.

## Authors’ contributions

BOA was involved in conceptualizing ideas, data analysis, interpretation of the results and writing of the manuscript. AA participated in the conception and design of the study, and reviewed the manuscript. P-OO helped in the design of the study, interpretation of the results and review of the manuscript. All authors read and approved the final manuscript.

## References

[B1] Boerma JT Bryce J Kinfu Y Axelson H Victora CG Mind the gap: equity and trends in coverage of maternal, newborn, and child health services in 54 Countdown countries Lancet 2008 371 9620 1259 1267 18406860 10.1016/S0140-6736(08)60560-7

[B2] Bhutta ZA Chopra M Axelson H Berman P Boerma T Bryce J Bustreo F Cavagnero E Cometto G Daelmans B Countdown to 2015 decade report (2000–10): taking stock of maternal, newborn, and child survival Lancet 2010 375 9730 2032 2044 10.1016/S0140-6736(10)60678-2 20569843

[B3] Hogan MC Foreman KJ Naghavi M Ahn SY Wang M Makela SM Lopez AD Lozano R Murray CJ Maternal mortality for 181 countries, 1980–2008: a systematic analysis of progress towards Millennium Development Goal 5 Lancet 2010 375 9726 1609 1623 10.1016/S0140-6736(10)60518-1 20382417

[B4] Lozano R Wang H Foreman KJ Rajaratnam JK Naghavi M Marcus JR Dwyer-Lindgren L Lofgren KT Phillips D Atkinson C Progress towards Millennium Development Goals 4 and 5 on maternal and child mortality: an updated systematic analysis Lancet 2011 378 9797 1139 1165 10.1016/S0140-6736(11)61337-8 21937100

[B5] Nations U: Department of E, Social A The millennium development goals report. 2010: 2010 2010 New York: United Nations, Department of Economic and Social Affairs

[B6] Bongaarts J Trends in unwanted childbearing in the developing world Stud Fam Plann 1997 28 4 267 277 10.2307/2137858 9431648

[B7] Gillespie D Ahmed S Tsui A Radloff S Unwanted fertility among the poor: an inequity? Bull World Health Organ 2007 85 2 100 107 10.2471/BLT.06.033829 17308730 PMC2636279

[B8] Stover J Ross J How increased contraceptive use has reduced maternal mortality Matern Child Health J 2010 14 5 687 695 10.1007/s10995-009-0505-y 19644742

[B9] Benagiano G Bastianelli C Farris M Contraception: a social revolution The European journal of contraception & reproductive health care : the official journal of the European Society of Contraception 2007 12 1 3 12 10.1080/13625180601012311 17455038

[B10] Conde-Agudelo A Belizan JM Maternal morbidity and mortality associated with interpregnancy interval: cross sectional study BMJ 2000 321 7271 1255 1259 10.1136/bmj.321.7271.1255 11082085 PMC27528

[B11] Zhu BP Rolfs RT Nangle BE Horan JM Effect of the interval between pregnancies on perinatal outcomes N Engl J Med 1999 340 8 589 594 10.1056/NEJM199902253400801 10029642

[B12] Rutstein SO Effects of preceding birth intervals on neonatal, infant and under-five years mortality and nutritional status in developing countries: evidence from the demographic and health surveys International journal of gynaecology and obstetrics: the official organ of the International Federation of Gynaecology and Obstetrics 2005 89 Suppl 1 S7 S24 10.1016/j.ijgo.2004.11.012 15820369

[B13] Cleland J Bernstein S Ezeh A Faundes A Glasier A Innis J Family planning: the unfinished agenda Lancet 2006 368 9549 1810 1827 10.1016/S0140-6736(06)69480-4 17113431

[B14] Creanga AA Gillespie D Karklins S Tsui AO Low use of contraception among poor women in Africa: an equity issue Bull World Health Organ 2011 89 4 258 266 10.2471/BLT.10.083329 21479090 PMC3066524

[B15] Shah IH Chandra-Mouli V Inequity and unwanted fertility in developing countries Bull World Health Organ 2007 85 2 86 10.2471/BLT.06.037366 17308725 PMC2636274

[B16] Marmot M Friel S Bell R Houweling TAJ Taylor S Commission on Social Determinants of H, Commission Social Determinants H: **closing the gap in a generation: health equity through action on the social determinants of health** Lancet 2008 372 9650 1661 1669 10.1016/S0140-6736(08)61690-6 18994664

[B17] Wirth ME Balk D Delamonica E Storeygard A Sacks E Minujin A Setting the stage for equity-sensitive monitoring of the maternal and child health Millennium Development Goals Bull World Health Organ 2006 84 7 519 527 10.2471/BLT.04.019984 16878225 PMC2627391

[B18] Zere E Kirigia JM Duale S Akazili J Inequities in maternal and child health outcomes and interventions in Ghana BMC Publ Health 2012 12 252 10.1186/1471-2458-12-252 PMC3338377 22463465

[B19] Bongaarts J Can family planning programs reduce high desired family size in Sub-Saharan Africa? Int Perspect Sex Reprod Health 2011 37 4 209 216 10.1363/3720911 22227628

[B20] Ezeh AC Bongaarts J Mberu B Global population trends and policy options Lancet 2012 380 9837 142 148 10.1016/S0140-6736(12)60696-5 22784532

[B21] Mathe JK Kasonia KK Maliro AK Barriers to adoption of family planning among women in Eastern Democratic Republic of Congo Afr J Reprod Health 2011 15 1 69 77 21987940

[B22] Blanc AK Tsui AO Croft TN Trevitt JL Patterns and trends in adolescents’ contraceptive use and discontinuation in developing countries and comparisons with adult women Int Perspect Sex Reprod Health 2009 35 2 63 71 10.1363/3506309 19620090

[B23] Hotchkiss DR Godha D Do M Effect of an expansion in private sector provision of contraceptive supplies on horizontal inequity in modern contraceptive use: evidence from Africa and Asia International journal for equity in health 2011 10 1 33 10.1186/1475-9276-10-33 21854584 PMC3171310

[B24] De Sherbinin A Spotlight: Ghana Population today 1993 21 7–8 11 12286891

[B25] Ghana Official Policy Statement Stud Fam Plann 1969 1 44 1 7

[B26] Government of Ghana National Population Policy (Revised edition 1994 At a glance 1994 Accra: National Population Council

[B27] Adanu RM Seffah JD Hill AG Darko R Duda RB Anarfi JK Contraceptive use by women in Accra, Ghana: results from the 2003 Accra Women’s Health Survey Afr J Reprod Health 2009 13 1 123 133 20687270

[B28] Ruthavth H Fronczak N Chinbuah A Miller R Ghana Trend Analysis for Family Planning Services, 1993, 1996, and 2002 2005 Maryland, USA: Calverton

[B29] Wagstaff A Paci P Van Doorslaer E On the measurement of inequalities in health Soc Sci Med 1991 33 5 545 557 10.1016/0277-9536(91)90212-U 1962226

[B30] Mackenbach JP Kunst AE Measuring the magnitude of socio-economic inequalities in health: an overview of available measures illustrated with two examples from Europe Soc Sci Med 1997 44 6 757 771 10.1016/S0277-9536(96)00073-1 9080560

[B31] Koolman AHE Socioeconomic Inequality in Health and Health Care: Measurement and Explanation. Ph.D. thesis 2006 Rotterdam, The Netherlands: Erasmus University Rotterdam

[B32] Moussa KM Ostergren PO Eek F Kunst AE Department of Laboratory Medicine L Are time-trends of smoking among pregnant immigrant women in Sweden determined by cultural or socioeconomic factors? BMC Publ Health 2010 10 1 374 10.1186/1471-2458-10-374 PMC2906466 20579380

[B33] Bambra C Popham F Worklessness and regional differences in the social gradient in general health: Evidence from the 2001 English census Health Place 2010 16 5 1014 1021 10.1016/j.healthplace.2010.06.006 20638320

[B34] Blanc AK Grey S Greater than expected fertility decline in Ghana: untangling a puzzle J Biosoc Sci 2002 34 4 475 495 10.1017/S0021932002004753 12395864

[B35] Ijaiya GT Raheem UA Olatinwo AO Ijaiya MD Ijaiya MA Estimating the impact of birth control on fertility rate in sub-Saharan Africa Afr J Reprod Health 2009 13 4 137 145 20690281

[B36] Mostafa Kamal SM Aynul Islam M Contraceptive Use: Socioeconomic Correlates and Method Choices in Rural Bangladesh Asia Pac J Public Health 2010 22 4 436 450 10.1177/1010539510370780 20659903

[B37] Tawiah EO Factors affecting contraceptive use in Ghana J Biosoc Sci 1997 29 2 141 149 10.1017/S0021932097001417 9881126

[B38] GSS Ghana Demographic and Health Survey 2008 2009 Accra, Ghana and Calverton, USA: MD: GSS, GHS, and ICF Macro

[B39] GSS Ghana Demographic and Health Survey 1988 1989 Accra, Ghana: Ghana Stastistical Services

